# Changes in physical activity levels and relationship to balance performance, gait speed, and self-rated health in older Swedish women: a longitudinal study

**DOI:** 10.1007/s40520-021-02016-5

**Published:** 2021-11-16

**Authors:** Marian E. Papp, Ann Charlotte Grahn-Kronhed, Hans Rauch Lundin, Helena Salminen

**Affiliations:** 1 Division of Family Medicine and Primary Care, Division of Physiotherapy, Department of Neurobiology, Care Sciences and Society, Karolinska Institute, Alfred Nobels alle 23, 141 83 Huddinge, Sweden; 2 Academic Primary Healthcare Centre Stockholm, Stockholm, Sweden; 3 Division of Physical Therapy, Department of Neurobiology, Care Sciences and Society, Karolinska Institute, Solna, Sweden; 4 Rehab Väst, Local Health Care Services in the West of Östergötland, Motala, Sweden; 5 Division of Prevention, Rehabilitation, and Community Medicine, Department of Health, Medicine and Caring Sciences, Linköping University, Linköping, Sweden

**Keywords:** Self-evaluation, Subjective-health, Falls, Balance, Postural physical exercise, Elderly, Women, Longitudinal design

## Abstract

**Background and aim:**

Physical activity levels in older people often decrease and may mean impaired physical functioning leading to an increased fall risk. The aim of this study was to investigate self-reported change in physical activity dose and deterioration in balance performance, gait speed, and self-rated health (SRH) in older women between two time points in a follow-up study.

**Methods:**

A cohort of community-living women, aged 69–79 years (*n* = 351) were evaluated by questionnaire and clinical tests on balance, gait speed, and SRH at baseline. One hundred and eighty-six women were followed-up by these tests 8.5 years after inclusion. The non-parametric Wilcoxon signed-rank test and Mann–Whitney *U* test were used for the analysis.

**Results:**

The greatest changes were seen in one-leg standing time (OLST) with eyes closed (− 60%) and eyes open (− 42%). The population was divided into high exercise (HE, *n* = 49) and low exercise (LE, *n* = 51) groups. At baseline the HE group had an OLST of 19 s with eyes open and 3 s with eyes closed. In the LE group, these values were 7.3 s and 2 s. At follow-up, differences between HE and LE concerning tandem walk forwards (steps) (HE = 8.5; LE = 2.5) and backwards (HE = 11; LE = 3.5) emerged. The HE group estimated SRH (VAS-scale) 30 mm higher at baseline and 17 mm higher at follow-up than the LE group.

**Conclusion:**

Greater physical activity seems to be an important predictor for maintaining physical function and SRH in older women.

## Background

Increased physical activity is associated with improvements in physical function such as balance ability and mobility in older adults [1, 2]. The current guidelines recommend moderate to vigorous intensity aerobic physical activity of at least 150 min per week to maintain functional abilities and health in older adults (> 65 years) [1]. Further, it is important to add muscle and bone strengthening activities that activate major muscle groups at least 2 days per week. Those with limited mobility should perform physical activities for health benefits and to enhance balance and prevent falls [2]. Balance and functional activities such as gait and one-legged movements may reduce fall rates and fall-related fractures in older people [3]. Balance performance as measured by one-leg standing time and walking speed is impaired by ageing in older women according to several studies [4–7] Moreover, objectively monitored physical activity has been shown to be associated with improved physical functioning, when the dose was increased at least 48 min per week [8]. Prospective studies are of importance, and there is lack of studies on how physical activity can contribute to healthy ageng in the older population.

Observational studies show that sarcopenia may be prevented by physical activity [9]. Sarcopenia involves generalized loss of skeletal muscle mass and muscle strength and can lead to physical disability, low quality of life, and death [10, 11]. Self-rated health (SRH) and the evaluation of gait speed and balance performance with static and dynamic tests are validated tests that were used in this longitudinal study [12–14]

The primary aim of this study was to explore changes in physical activity levels and functional tests such as dynamic and static balance ability, gait speed, and also SRH during a longitudinal follow-up in older women. Another aim was to evaluate relationship between physical activity levels and the functional tests.

## Materials and methods

### Population and data collection

**Fig. 1:**
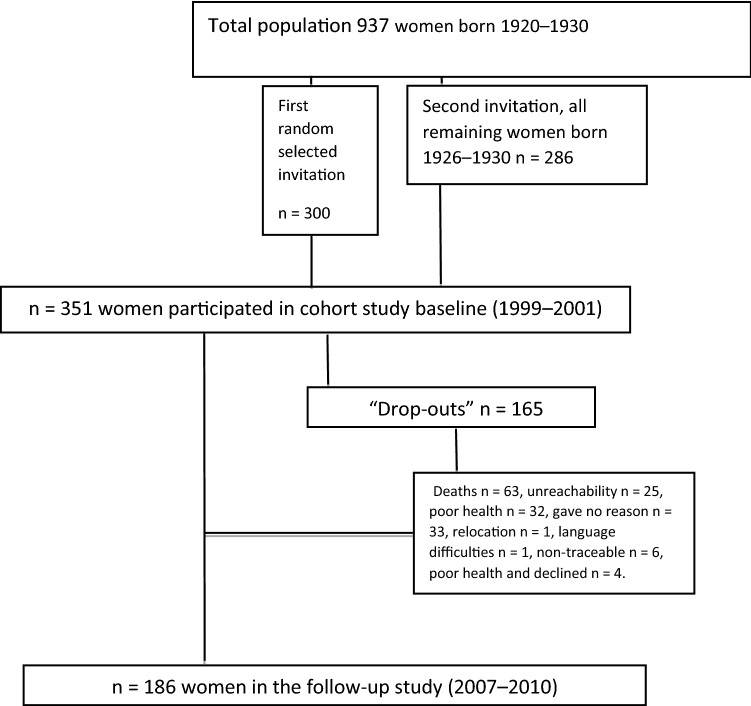
Flow chart for the baseline and the follow-up of the study

A cohort of community-living women, aged 69–79 years (median age 72.4 years) at baseline, were followed with analyses of gait speed, balance ability, and physical activity dose in a longitudinal study (1999–2010). The women were part of the PRIMOS project (PRIMary health care and OSteoporosis) [15]. Inclusion criteria for participation in the study were being a woman born between 1920 and 1930, and residing in a southern suburb of Stockholm in Sweden. Of the 937 eligible women, 586 were sent written invitations and 351 women agreed to participate at baseline (1999–2001) [15]. One hundred and eighty-six women were followed-up after a mean of 8.5 years (median 8.2 years, range 7.9–10.7 years) (2007–2010) in the longitudinal study (Fig. 1). During the follow-up, 165 of the original 351 participants (Fig. 1) dropped out due to unreachability, illness, death, relocation, or declining to participate. During follow-up 63 persons had died and the 102 surviving non-participants had a median age of 81 years (Fig. 1). Mortality data were obtained on the individual level from the Swedish National Board of Health and Welfare in 2010.

### Measurements

The participants included in this longitudinal study answered a questionnaire and performed tests of functional ability both at baseline and at follow-up (Fig. 1). Further subgroup analysis was performed with participants divided into high and low exercise doses to determine if the amount of exercise was associated with the physical function tests.

To be included in the study the participants had to be able to visit the primary health care center for the examinations. At the follow-up, home visits were also offered if the participant had limited mobility. At baseline, the same physician collected all data and examined the participants to prevent biased measurements. At the follow-up, the same physician together with a doctoral student collected data from all the examinations.

### Physical function tests

Participants performed several physical function tests such as gait speed, static and dynamic balance, and chair-stand tests. For the gait speed test, the participants wore shoes and walked in a corridor as fast as they could for a total of 30 m (15 m with a turn without losing their balance, walking aids were allowed). The total time was recorded and gait speed in m/s was calculated. Gait speed is a validated measure for self-selected and maximum speed [13]. A cut-off point of gait speed ≤ 0.8 m/s is indicative of risk of sarcopenia [16].

The static balance tests were performed without shoes with the eyes open and with the eyes closed and the arms held crossed in front of the body. The floor was leveled and the room was well illuminated. During the OLST (one-leg standing time) tests the participants performed two tests on each leg for up to a maximum of 30 s. If the participant had contact with the floor with the non-standing foot the time was stopped. The two attempts were recorded using a digital stop-watch and the mean OLST was calculated. OLST has in previous studies been shown to have an intra-class correlation coefficient between 0.6 and 0.75 for inter-rated reliability and 0.95 for test–retest reproducibility [14, 17, 18].

The dynamic balance tests were tandem steps forwards (heel to toe) on a line and tandem steps backwards between two lines (toe to heel). Each test was performed twice and maximized to 15 accurate steps. The mean of two attempts was used for the analysis. The balance tests are established and reliable measurements [18–21]

The chair-stand test was performed with the arms crossed in front of the chest. A dichotomous variable was registered for being able or unable to rise once from a 45 cm high chair [22].

### Physical activity level

Current physical activity level was self-reported using two questions. The first question was, “How often do you perform physical activity in any form each week, for example walking or gymnastics?” The question had four response alternatives: (1) less than once a week, (2) 1–2 times a week, (3) 3–4 times a week, and (4) 5 times or more per week. The second question was, “How long do you perform walking or gymnastics each day?” Three alternatives were available: (1) less than 15 min, (2) 15–30 min, and (3) more than 30 min.

In the subgroup analysis, we defined physical activity of more than 30 min per day at least 5 times/week as high exercise (HE) according to current exercise recommendations of at least 150 min physical activity per week [23], and physical activity of 30 min or less per day 1–4 times/week was defined as low exercise (LE) (meaning all the others in the sample).

### Self-rated health by visual analogue scale

Self-rated health (SRH) was estimated using a visual analogue scale (VAS) [24], which has been used for estimating quality of life, mood, well-being, and psychological pain. The VAS consists of a horizontal 100 mm line with the end-points of 0 for the worst imaginable health and 100 for the best imaginable health, and the individual marks their perceived health on the line [12]. The VAS was initially used in psychology for the measurement of mood disorders and for the measurement of pain [25], and it has a test–retest reliability of 0.8 for pain measurements [26]. Recently reported cut-offs for SRH in the age group in the present study were as follows: low SRH = 5–51 mm, intermediate SRH = 52–73 mm, and high SRH = 74–99 mm [12].

### Frequency of falls

At the follow-up the participants answered a question about the frequency of falls in the past year. There were three alternatives—(1) no falls, (2) 1 or 2 falls, and (3) 3 or more falls.

### Bone density measurements

The WHO released the first guidance (1994) for using bone mineral density (BMD) measurements to diagnose osteoporosis. When standard units are used in relation to BMD in young healthy women, this is referred to as the *T* score using Dual-energy X-ray absorptiometry (DXA) equipment at the lumbar spine or the hip.

Cut-off values for normal BMD are a *T* score above − 1 SD, while a *T* score between − 1 and − 2.5 SDs indicates osteopenia and a score of ≤ − 2.5 SD is the cut-off for the diagnosis of osteoporosis. Established osteoporosis is a condition with a *T* score ≤ − 2.5 combined with a fragility fracture. The BMD of the lumbar spine and the hip was measured using a Hologic QDR 4500 DXA equipment (Hologic Inc., Waltham, MD, USA).

### Statistics

Statistical analysis was performed using the non-parametric Mann–Whitney *U* test for the differences between groups and the Wilcoxon signed-rank test for matched measurements. The Shapiro–Wilk method was used to test for normality. Relative changes in each individual were calculated within the HE and LE groups, and median differences were calculated between the groups at baseline and at follow-up. *p* values < 0.05 were considered significant. All analyses were performed using STATA 14 (Stata Corp, TX, USA).

### Ethical considerations

Ethical approval was obtained from the Ethical Review Board of Stockholm at baseline (1998/145/98) and for the follow-up (2007/188-31/3). Written and oral informed consent was collected from all participants prior to enrolment. All data were treated in accordance with the Swedish Personal Data Act.

## Results

In this longitudinal study of 8.5 years, the median age of the participating women was 72.4 years at baseline (*n* = 351) and 81 years at follow-up (*n* = 186). The median for SRH was 62 mm (5–99) at baseline and 67 mm (6.7–99) at follow-up (Table 1). Relative changes between baseline and follow-up in the different tests were greatest for the OLST—right leg with eyes closed (− 60%) followed by the left leg with eyes open (− 42%) and left leg with eyes closed (− 40%). At baseline a total of 88% of the women were able to rise once from a chair without assistance of the arms, and at follow-up 83% were able to rise from a chair (Table 2).

**Table 1:** Characteristics at baseline and follow-up in a cohort of older women

Parameter	Baseline	Follow-up
Number of participants	*n* = 351	*n* = 186
Age years, median (range)	72.4 (68.5–79.2)	81 (77–87)
Weight kg, median (range)	69 (45–126)	67 (42.8–110)
Height cm, mean (SD)	161.8 (± 5.9)	159.8 (± 6.2)
BMI kg/m^2^ median (range)	26.1 (16.8–47.9)	26.3 (17.8–42)
Self-rated health VAS (100 best perceived health) mm, median (range)	62 (5–99)	67 (6.7–99)
No smoking habits, *n* (%)	198 (57%)	117 (63%)
Current smoker, *n* (%)	54 (15%)	19 (10%)
Former smoker, *n* (%)	99 (28%)	50 (27%)
Number of participants taking more than three medications, *n* (%)	265 (75%)	83 (45%)
Femoral neck *T* score mean (SD)	− 1.8 (± 0.9)^a^	− 1.8 (± 0.9)^b^
Osteoporosis, *n* (%)	76 (22%)^a^	33 (23%)^b^
Participants managing one Chair-stand test, *n* (%)	313 (88%)	155 (83%)

*BMI* body mass index, *SD* standard deviation; number of subjects measured at the femoral neck site, ^a^*n* = 340, ^b^*n* = 143

**Table 2:** Change in physical function tests between baseline and follow-up (all balance tests deteriorated)

Physical function test	Median change (range) baseline—follow-up	Relative change %	*p* value
Gait speed time for 30 m, s^a^	4.5 (− 76, 50)	20	< 0.001*
Gait speed m/s^b^	0.3 (− 0.4, 1.3)	18	< 0.001*
OLST Right leg, eyes open, s^c^	− 4.5 (− 17, 26.5)	35	< 0.001*
OLST Left leg, eyes open, s^c^	− 7 (− 11.5, 28)	42	< 0.001*
OLST Right leg, eyes closed, s^d^	− 1.5 (− 5, 24)	60	< 0.001*
OLST Left leg, eyes closed, s^e^	− 1 (− 3, 17.5)	40	< 0.001*
Tandem steps forwards^f^	− 4 (− 7, 15)	32	< 0.001*
Tandem steps backwards^f^	− 4 (− 8.5, 15)	27	< 0.001*

^a^*n* = 164; ^b^*n* = 160; ^c^*n* = 179, ^d^*n* = 171; ^d^*n* = 168; ^e^*n* = 173; ^f^*n* = 165; *p value calculation Wilcoxon signed-rank test; OLST = one-leg standing time

The 30 m gait-speed time deteriorated 4.5 s from baseline to follow-up (mean gait speed change between baseline and follow-up was 0.3 m/s). Table 2 shows the median in the 30 m gait speed time between the two measurements. In the within group analyses, all tests showed significant changes between the two time points (Table 2).

Forty-nine participants reported HE, while 51 participants reported LE at both time points. Table 3 illustrates the comparisons between HE and LE at baseline and at follow-up (the number of participating individuals varied in the tests).

**Table 3:** Difference in gait speed, balance performance, and self-rated health between high exercise (HE) and low exercise (LE) groups at baseline and at follow-up

	Difference between HE^a^ and LE^b^ at baseline		Difference between HE^a^ and LE^b^ at follow-up	
		*p* value		*p* value
Gait speed time for 30 m, s	3	0.01*	3	0.19
Gait speed m/s	0.38	0.02*	0.3	0.44
OLST Right leg, eyes open, s	11.7	0.01*	5	0.07
OLST Left leg, eyes open, s	13.5	< 0.001*	4	0.06
OLST Right leg, eyes closed, s	1	0.05	0.3	0.10
OLST Left leg, eyes closed, s	1	0.01*	0.5	0.07
Tandem steps forwards	5.5	0.09	6	< 0.001*
Tandem steps backwards	6	< 0.001*	7.5	< 0.001*
Self-rated health	29.5	< 0.001*	17	0.03*

^a^*n* = 49; ^b^*n* = 51; *significant *p* value < 0.05; OLST = one-leg standing time

High exercise group = physical activity 5 times/week or more for more than 30 min each time. Low exercise group (all others) = physical activity 1–4 times/week or less for 30 min or less each time

Analysis between the two exercise groups showed significant differences in most balance tests at baseline, favoring the HE group (Table 3).

Gait speed (*p* = 0.01), OLST with the right leg and eyes open (*p* = 0.01), OLST with the left leg and eyes open (*p* < 0.001), and OLST with the left leg and eyes closed (*p* = 0.01) were significantly different between the HE and LE groups at baseline. There was also a significant difference between the groups concerning the number of tandem backwards steps with 15 steps in the HE group and 9 steps in the LE (*p* < 0.001). At follow-up, there were differences between the groups regarding tandem forwards and backwards steps (*p* < 0.001) (Table 3).

The differences in SRH, as measured with the VAS, were statistically significant between the HE and LE groups both at baseline (*p* < 0.001) and at follow-up (*p* = 0.03), where the HE group estimated their SRH to be 30 mm higher at baseline and 17 mm higher at follow-up compared to the LE group (Table 3).

The relative changes compared to baseline were similar in both exercise groups regarding the physical function tests, and all balance tests deteriorated by 17–50% in the HE group and by 18–56% in the LE group (Table 4).

**Table 4:** Change and relative change in gait speed, balance performance, and self-rated health within high exercise (HE) and low exercise (LE) groups at baseline and at follow-up

	High exercise				Low exercise			
	Baseline Median (range)	Follow-up Median (range)	Relative change %	*p* value	Baseline Median (range)	Follow-up Median (range)	Relative change %	*p* value
Age years (median, range)	72 (69.1–78.6)	80.3 (77.1–86.7)^a^		NA	73 (69.8–78.3)	81.4 (78.1–86.3)^b^		NA
Gait speed time for 30 m, s	19 (14–42)	24 (16–61)	21	**< 0.001**^**a**^	22 (15–50)	27 (0–97)	18	**< 0.001**^**c**^
Gait speed m/s	1.58 (0.71–2.14)	1.2 (0.11–1.88)	17	**< 0.001**	1.5 (0.68–2.14)	1.2 (0.31–2.00)	19	**< 0.001**^**c**^
OLST Right leg eyes open, s	19 (1.5–30)	7.5 (0–30)	37	**< 0.001**^**d**^	7.3 (0–30)	2.5 (0–30)	45	**< 0.001**^**b**^
OLST Left leg eyes open, s	22.5 (2–30)	7 (0–30)	44	**< 0.001**^**d**^	9 (0–30)	3 (0–30)	39	**< 0.001**^**b**^
OLST Right leg closed eyes closed, s	3 (0–30)	1.3 (0–7)	50	**< 0.001**^**a**^	2 (0–11)	1 (0–10)	50	**< 0.001**^**b**^
OLST Left leg eyes closed, s	3 (0–21.5)	1.5 (0–6.5)	50	**< 0.001**^**a**^	2 (0–7)	1 (0–4.5)	50	**< 0.001**^**b**^
Tandem steps forwards	14.5 (2.5–15)	8.5 (0–15)	21	**< 0.001**^**a**^	9 (0–15)	2.5 (0–15)	44	**< 0.001**^**c**^
Tandem steps backwards	15 (2.5–15)	11 (0–15)	22	**< 0.001**^**a**^	9 (0–15)	3.5 (0–15)	56	**< 0.001**^**c**^
Self-rated health	80 (21–99)	72 (6.7–99)	6	**0.05**^**a**^	50.5 (6–94)	55 (13–89)	8	0.06^b^

^a^*n* = 49; ^b^*n* = 51; ^c^*n* = 41; ^d^*n* = 45; OLST = one-leg standing time; *significant *p* value < 0.05. Wilcoxon signed-rank test for matched pairs. High exercise group = physical exercise 5 times/week or more for more than 30 min each time. Low exercise group (all others) = physical exercise 1–4 times/week or less for 30 min or less each time. Relative changes are compared to baseline values

The self-rated number of falls was registered at the follow-up, and 107 women reported no fall during the past year, 66 reported one or two falls, and 12 reported more than two falls. In the HE group, 26 individuals (53%) reported at least one fall in the past year at the follow-up, and in the LE group 51 individuals (100%) reported at least one fall.

Regarding the chair-stand test, two individuals in the HE group could not rise from the chair at baseline and these subjects and another six individuals could not rise at follow-up. In the LE group, 11 individuals could not rise from chair at baseline and these subjects and another 11 individuals could not rise at follow-up. There were eight women in the LE group and four in the HE group who reported a hip fracture during the follow-up period.

## Discussion

In this longitudinal follow-up in older women, the primary aim was to explore change in physical activity levels and the performance of functional tests such as dynamic and static balance ability, gait speed, and also SRH. In addition, we evaluated the relationships between physical activity levels and the functional tests.

### Gait speed

The median change in 30 m gait speed time between baseline and follow-up was 4.5 s slower and 0.3 m/s slower at the follow-up. Deteriorating walking speed in older women while ageing is in agreement with other studies [4, 7]. However, the women in our sample who were about 70 years at inclusion had faster gait speed (1.5 m/s) than the normative value for this age group [6].

Normative reference values for gait speed are 1.24 m/s for women in the age group 60–69 years, 1.13 m/s for the age group of 70–79 years, and 0.94 m/s for the age group 80–99 years [6]. In a 7-year follow-up study of healthy women (mean age 80.5 years), it was found that the time required to walk 30 m was increased from 20.9 s to 23.1 s. In that study, the mean walking speed was 1.30 m/s for women [4]. Lundgren-Lindquist et al. found in a population study of 79-year-old women that maximum walking speed was 1.18 m/s [27]. Slower walking speed with increased age is influenced by both mental and physiological parameters, and fear of falling usually affects both gait speed and postural patterns of movement [28]. A cut-off of 1.0 m/s for gait speed was reported to be useful for identifying those vulnerable to falls among women in their late 70 s, and that study showed that a low gait speed was associated with a history of multiple falls [29]. A concern about falling usually leads to greater alterations in walking speed and step length as well as to doubling the support time of the walking cycle. Gait speed is a highly validated test that can predict a decline in mobility and death [13], and it is a robust measure of functional status and overall health [30]. Cummings et al. reported that poor performance on gait speed is associated with an increased risk of hip fracture [31]. Gait speed is also an acceptable predictor of hip fractures independent of 10-year fracture risk probability assessed by FRAX [32].

### Balance

The balance test results deteriorated by age in our sample, which is in accordance with other studies [33]. The normative mean value for OLST with eyes open is 25.1 s in women aged 60–69 years, and this decreases to 11.3 s in women aged 70–79 years and further decreases to 7.4 s in women aged 80–99 years [33]. The values for OLST in women in the present study significantly deteriorated between baseline and follow-up within both the HE and LE groups. In our sample, there was a difference between the two exercise groups, favoring the HE group.

The OLST with eyes open decreased by 37–45% and the OLST with eyes closed decreased by 50% in both the HE and the LE groups at follow-up compared to baseline. A previous 7-year follow-up study of healthy older persons showed that balance was significantly impaired in OLST with the eyes open and with the eyes closed [4]. A review showed that mean normative values for OLST with eyes closed were 2.2 s in the age group 70–79 years and 1.4 s in the age group 80–99 years [33]. Older persons tend to rely more on the eyes while balancing, because proprioception and vestibular information are decreasing with age, which is mirrored in the results for the OLST with eyes closed [33, 34]. Previously it was shown that an OLST with eyes open of less than 10 s in older women was associated with an almost threefold greater risk for hip fracture compared to those who managed to balance for 10 s or more. It was also shown that a 1 s longer OLST balance time with eyes open decreased the risk for a hip fracture [35, 36].

Regarding the LE and HE groups, we compared self-reported physical activity levels and found that significant changes emerged within the groups with deteriorated results with age in all functional balance tests. The differences between LE and HE regarding balance tests and SRH at baseline were all significant (except the tandem forward line steps), favoring the HE group. Regarding differences between the groups at the follow-up, less significance appeared, but tandem forwards and backwards steps were significantly different as well as SRH.

### Chair-stand, osteoporosis, falls, and fracture

Chair-stand and gait speed are both part of physical performance tests often used for the diagnosis of sarcopenia [16]. Sarcopenia is a geriatric syndrome and is diagnosed as low muscle mass and low muscle strength [37–39]. Chair rising ability is strongly associated with lower extremity muscle strength, and the chair-stand is an important test to evaluate muscle strength and neuromuscular function. In our sample more than 80% of the participants were able to rise once from a chair without support both at baseline and at follow-up. Poor muscle strength has been reported to have a relative risk of 1.3–3.2 for hip fracture [31, 40]. In this sample we did not measure the number of falls at baseline, but a single self-rated question about the frequency of falls in the past year was asked at the follow-up. The majority (*n* = 107) in our sample reported no falls during the past year. When the models included the inability to rise from a chair without using the arms, no other measurement of neuromuscular function remained significantly associated with the risk of hip fracture [41]. In the LE group, all individuals reported at least one fall during the past year, and in the HE group half of the individuals (53%) reported at least one fall. The total number of falls was greater in the LE group and the ability to rise from a chair was worse in LE group, and this suggests that the LE group had less muscle strength and worse balance compared to HE group. However, it is important to note that the number of falls was self-reported. Balance tests, self-reported fall history, and gait speed are acceptable predictors of hip fractures according to the Osteoporosis Prospective Risk Assessment study [42].

Osteoporosis was seen in 22% of the women at baseline and in 23% at follow-up. This means that many women in our sample were at risk for hip fracture, because there was a total of 12 women in the sub-groups reporting a hip fracture during the follow-up period. In the Million Women prospective study, it was shown that increased frequency of strenuous activity and any exercise was associated with a 37% lower risk of self-reported hip fractures [43].

### Self-rated health (SRH)

The participants in our sample estimated their SRH as intermediate (ranging between 52 and 73 mm). The VAS rating showed that the HE group estimated their SRH higher both at baseline and at follow-up compared to the LE group. This indicates that exercising is associated with better perceived health.

Those women reporting better SRH seemed to perform the recommendation of at least 150 min physical activity per week. The change in SRH was less than 10 mm between baseline and follow-up in both the HE and the LE groups.

Also, ethnicity, gender, and age may vary regarding SRH [44]. Associations between higher self-rated health and physical activity among older adults is clear [45, 46]. New data support that there might be an association between hip fracture and SRH [12].

### Physical activity for older adults

A Swedish study found that increased physical activity is beneficial for health, especially if sedentary time is replaced with light-intensity physical activity [47]. New data regarding function and disability show increased prevalence of frailty and decreased function with sedentary lifestyle [48]. Walking is probably one of the best forms of physical activity for older adults [49]. The number of steps per day affects balance, and a Swedish study in community-dwelling women aged 66–86 years showed that participants taking fewer than 5000 steps per day had slower gait speed, poorer balance performance, and lower health-related quality of life compared to participants with 5000 steps or more per day. In that study the OLST was twice as long in the high steps group (8.85 s) compared to the low steps group [50]. Habitual daily walking activity at 1 m/s and taking at least 5000 steps/day is associated with maintained proximal femur bone mineral density (*T* score) in healthy middle-aged women with normal body weight [51]. Thus, at least 5000 steps per day seems to be an important cut-off for good balance and gait speed for optimal overall health and physical function in older adults. However, in the present study we did not measure the number of habitual steps.

A recent review [23] on persons aged over 65 years showed that physical activity interventions may improve bone health (low to moderate evidence) and thus prevent osteoporosis. The level of evidence is somewhat higher for lumbar spine BMD compared to femoral neck. The exercise dose where significant effects appeared was 60 min or more two or three times a week for a duration of seven months [23]. It has also been suggested that multicomponent programs that combine impact exercise and progressive resistance training at a moderate or high intensity are the most effective [52]. For general health, daily balance exercises are important for older adults as well as aerobic exercise, spine-caring exercises, and posture awareness training [52].

Individuals with osteoporosis are recommended to engage in multicomponent exercise training such as resistance, strength, balance, and three-dimensional training, for instance dance and Tai Chi [53]. Also, high-speed training combined with simulated functional tasks may be used to enhance functional outcomes [41].

### Strengths and limitations:

A limitation of the present study is that it included a rather small sample size and only women. However, focusing on women is also a strength of this study since physical function trajectories might differ based on gender. Another limitation is that we did not extend the analysis with more covariates as the sub-groups, which relied on reported physical activity dose. Residual confounding cannot be fully discarded given the potential heterogeneity in subclinical health states and lifestyle factors.

Self-reported physical activity is not the optimal indicator for determining physical activity level. In this study, we did not use any pedometer, which is a more validated measurement of performed physical activity.

A more validated measure than our single chair-stand test is using time and counting the number of rises from the chair, which is a good measure of lower extremity muscle strength.

The strengths of the present study are the longitudinal design with an average follow-up period of 8.5 years and a sample of population-based older women. Another strength is the number of validated balance tests used in the present study, where several balance tests are part of the validated Berg’s balance scale battery.

## Conclusion

Balance performance and gait speed deteriorated in older women over the follow-up period of about 8.5 years. Balance ability by OLST, tandem backwards, and gait speed differed between the HE and LE groups at baseline. At the follow-up the tandem forwards and backwards balance tests were significantly different between the groups. A high physical activity level might be important to maintain perceived health in older women.
